# The Care-Dependent are Less Averse to Care Robots: An Empirical Comparison of Attitudes

**DOI:** 10.1007/s12369-023-01003-2

**Published:** 2023-05-29

**Authors:** Manuela Schönmann, Anja Bodenschatz, Matthias Uhl, Gari Walkowitz

**Affiliations:** 1 Faculty of Computer Science, Technische Hochschule Ingolstadt, Ingolstadt, Germany; 2 TUM School of Social Sciences and Technology, Technical University of Munich, Munich, Germany; 3 Seminar for Corporate Development and Business Ethics, Faculty of Management, Economics and Social Sciences, University of Cologne, Cologne, Germany; 4 Faculty of Business Administration, Technische Universität Bergakademie Freiberg, Freiberg, Germany

**Keywords:** Care robots, Nursing care, Robot aversion, Affective attitudes, Vignette experiment

## Abstract

A growing gap is emerging between the supply of and demand for professional caregivers, not least because of the ever-increasing average age of the world’s population. One strategy to address this growing gap in many regions is the use of care robots. Although there have been numerous ethical debates about the use of robots in nursing and elderly care, an important question remains unexamined: how do the potential recipients of such care perceive situations with care robots compared to situations with human caregivers? Using a large-scale experimental vignette study, we investigated people’s affective attitudes toward care robots. Specifically, we studied the influence of the caregiver’s nature on participants’ perceived comfort levels when confronted with different care scenarios in nursing homes. Our results show that the care-robot-related views of actual care recipients (i.e., people who are already affected by care dependency) differ substantially from the views of people who are not affected by care dependency. Those who do not (yet) rely on care placed care robots’ value far below that of human caregivers, especially in a service-oriented care scenario. This devaluation was not found among care recipients, whose perceived level of comfort was not influenced by the caregiver’s nature. These findings also proved robust when controlled for people’s gender, age, and general attitudes toward robots.

**Supplementary Information:**

The online version contains supplementary material available at 10.1007/s12369-023-01003-2.

## Introduction

In many regions of the world, an ever-increasing demand for support from long-term care services is confronted with a shrinking number of professional caregivers [1, 2]. Currently, many people in need of care are cared for at home, mostly by female family members as informal caregivers [3]. However, changes in gender roles, decreases in family size and broadening geographic dispersal have increasingly limited families’ ability to care for their relatives [3]. Additionally, rising life expectancies and falling birth rates are leading to an ongoing demographic shift [1, 2]. As older age is usually accompanied by physical decline and health problems, this demographic shift has increased the number of people with health conditions who need help from others to perform activities of daily living^1^ (i.e., care dependency) [3]. At the same time, however, the number of new entrants into the nursing profession is falling [5], and employees often leave the profession early due to working conditions that cause high physical and psychological stress [6]. The availability of specialized caregivers is, therefore, increasingly insufficient in many regions of the world [2, 7].

One solution to address the increasing gap between demand for and supply of professional caregivers could be the use of assistive technologies and robotics to supplement human caregiving. Care robots (i.e., “robots intended to assist or replace human caregivers in the practice of caring for vulnerable persons such as the elderly, young, sick, or disabled” [8, p. 251]) are often presented as an attractive technological solution for mitigating the problems incurred by structural societal and demographic change and for alleviating the shortage of nursing staff [9]. However, in recent years, there have been numerous debates about the use of robots in nursing and elderly care. The scenarios discussed range from very optimistic visions of the future, in which care robots are new family members who are fully integrated into society [10], to extremely negative dystopias in which robotic devices represent the decline of humane and empathic care [11, 12]. The latter include concerns that care robots could increase care recipients’ social isolation, reduce their well-being, and violate their dignity [13–16]. If human attention, human caring, and compassion are seen as the core of care activities for the elderly and sick [17], robots by their very nature cannot provide “genuine care,” but only a “behavioral mimicry of ‘care’” [18]. In this view, the use of robots would remove the “human element” from care [18], which could then be reduced to purpose-driven concerns under economic efficiency pressure [19]. In contrast, the use of robotic devices could also improve the autonomy of people in need of care [20]. For instance, by reducing dependence on others for basic activities of daily living (such as eating, bathing or toileting), care robots could help people in need of care to meet basic human needs independently [18, 20]. Shifting routine tasks to robots could also allow human caregivers to focus more on emotional and interpersonal aspects of care [17]. Thus, the use of care robots could also positively affect the sense of dignity and well-being of people in need of care [16, 20, 21].

### Purpose of the Present Research

The question of whether we should welcome or reject care robots in our society has received considerable attention from a normative perspective. Moreover, care robot acceptance is often evaluated in very general terms according to whether people can *imagine being cared for by robots* and on the premise that the use of robots could prevent a move to a nursing home (see, for instance, 22–24). Studies on technologies of what is called ambient assisted living (or, in the Nordic countries, welfare technologies) have more specifically investigated people’s acceptance of assistive technologies and care robots for home use (e.g., with regard to the needs of elderly people, possible ethical issues and emotions triggered by the technology) [25–28]. Strictly speaking, however, these studies do not measure the acceptance of care robots in relation to the acceptance of human caregivers, but implicitly compare the scenarios of *receiving care from a human but having to move to a nursing home* to that of *being able to live at home but receiving care from a technical device*. Much of the existing literature has also addressed conceivable applications and tasks for social and care robots, as well as the influence of certain design features and characteristics on people’s acceptance of particular care robots (see, e.g., 22, 23, 29–33). Attitudes toward social robots (including care robots), in terms of acceptance, fear, affective, cognitive, and behavioral attitudes, have also been the subject of empirical research with varying results (see, e.g., 23, 34). While some studies have concluded that people have rather positive attitudes toward social robots [22–25, 35] and would not be averse to interacting with them [34], others have found more mixed reactions [9, 36] or categorical aversion toward robots, especially in the area of care for the elderly, the disabled and the very young [37–40].

However, the important question of how people perceive care scenarios with care robots *compared* to exactly the same situations with human caregivers^2^ has remained, to our knowledge, unexplored. A research gap therefore arises in terms of how people’s affective attitudes (i.e., their feelings or emotions) toward care robots *compare* to their affective attitudes toward human caregivers, particularly in the context of nursing homes.

Using a large-scale experimental vignette study, we therefore set out to investigate the following research question: What influence does the caregiver nature have on people’s affective attitudes, measured in terms of perceived comfort level? To this end, we confronted a sample with different care scenarios in nursing homes. To our knowledge, there is currently no care robot capable of providing the same level of care as a human caregiver is. Therefore, a study using currently available care robots could only provide product-specific results for a very limited range of applications, precluding a comparison of affective attitudes toward care robots and human caregivers. For this reason, we decided to use the vignette study method, which has long been used in social sciences and nursing research [41] and is considered “a valuable technique for exploring people’s perceptions, beliefs and meanings about specific situations” [42]. A strength of the vignette approach is its use of respondents’ reactions to hypothetical situations and potential to elicit their attitudes toward situations beyond their own current circumstances. With this approach, such reactions are less influenced by social desirability, especially for sensitive topics, than are more direct self-reports [43, 44]. In addition, the controllability of the experimental manipulation (i.e., the changes in the independent variables) allows accurate inferences to be made about the extent to which these manipulations influence respondents' attitudes or intentions [43]. Following van Wynsberghe [45], we define care robots according to their field of application, intended use, and intended users. We do not limit our definition to a particular appearance or type of robot but consider care robots as a general category of robots that replace human caregivers in nursing homes’ sphere of daily care activities for individuals in need of care, both for intimate care tasks in the narrow sense (such as helping with personal hygiene) and for non-intimate, more service-oriented tasks (such as carrying and bringing things).

As outlined above, the development of robots able to perform caregiving tasks is often rejected in public discourse as inhumane and inappropriate. Our study contributes to the discourse on the acceptability of care robots by providing important insights into whether hypothetical care recipients (i.e., people who might be affected by care dependency later in life) and actual care recipients (i.e., those who are already in need of care) share the same views. A strength of our study lies in its quantitative nature. By assessing participants’ affective attitudes in a quantitative manner, we are able to concretely illustrate the differences in attitudes of actual and hypothetical care recipients when confronted with care robots. In contrast to other authors who, for example, examine the acceptance of care robots using the concept of human dignity [16], their influence on elderly people’s sense of autonomy [46] or personality traits’ effect on elderly people’s change in attitude toward robots [47], we took a hedonic approach. By asking people how comfortable they would feel in different care scenarios, we aimed to obtain their non-rationalized, emotional, perception of these situations and the respective caregivers. Emotional reactions and feelings toward things, ideas, or other people, are referred to as *affective attitudes* (as opposed, e.g., to cognitive attitudes, which include a person's thoughts and beliefs) [48, 49]. Attitudes and feelings such as anxiety have been shown to have a significant impact on older people's intention to use assistive robot technology [50]. Affective attitudes can, therefore, also be considered an important factor in the acceptance of potential care technologies. Furthermore, we analyze affective attitudes toward care robots *compared* to affective attitudes toward human caregivers, thus comparing respondents’ perceptions of a potential technology to those of an actual state. A better understanding of the way in which people, especially those who are already affected by care dependency, perceive potentially useful care technologies could help alleviate the problem of caregiver shortages in socially and demographically dynamic societies.

In the following section, we give an overview of related work. Section 3 describes the methods used in our study, including our research hypotheses, the study and vignette design, the measures, and information on the participants and procedure. In Sect. 4, we present the study results, of which the implications are discussed in Sect. 5, and the final section provides the conclusion.

## Related Work

### General Attitudes Toward Care Robots

In surveys about their attitudes toward care robots, people are often asked in general terms whether they could *imagine being cared for by robots*. Especially under the premise that this would enable them to live longer in their homes in old age instead of moving into a nursing home, many respond in the affirmative [22, 23]. Studies in different fields of social science and health care have indicated differences in attitudes depending on the population surveyed, the context of use, and prior exposure to robots [29, 35]. For example, in their literature review of robots in various occupational settings, Savela et al. [35] found that studies in which participants were exposed to actual robots were more likely to identify overall positive attitudes toward them than studies with hypothetical robots were. In their systematic literature review of attitudes toward social robots in general, Naneva et al. [34] concluded that people tend to have mildly positive attitudes toward social robots and would not be averse to interacting with them should the opportunity arise. Consistent with this, participants in a study by Hoppe et al. [51] showed a preference for a caregiver robot when asked to choose between a human and a robotic caregiver for assistance in daily living. When evaluating findings about perceptions and attitudes toward the specific use of assistive robots for the elderly, however, Plaschka et al. [36] found very mixed reactions, with only one of the studies included in their scoping review finding no overall negative attitude toward or rejection of assistive and care robots. While such studies’ participants perceived reduced dependence on caregivers or family members (and, thus, increased autonomy for elderly robot users) as positive, negative responses often related to safety concerns, privacy or responsibility issues, and ethical considerations [36, 52]. The latter include concerns that care robots could socially isolate older people, limit their self-determination, threaten their self-efficacy, objectify them, deprive them of social recognition, and violate their dignity [11, 14, 23, 52, 53].

The Eurobarometer, a large-scale survey of nearly 28,000 respondents in EU member states, has shown that EU citizens generally have a positive attitude toward robots and see them as a good thing for society, as “they help people do their jobs or carry out daily tasks at home” [38]. However, the proportion of respondents with a positive attitude toward robots has been declining since 2012, indicating a clear negative trend in public opinion toward robots [54]. Also, most respondents would not be comfortable with robots providing services and companionship for older people, nor with their use in caring for children, and the disabled, believing that robots should be banned from these areas of life altogether [37–39]. A survey by the non-partisan Pew Research Center revealed that U.S. respondents shared this rather negative attitude toward care robots: Most respondents said they would not be interested in using a care robot for themselves or a family member if they had the option. The most frequently cited reason was that the use of care robots would reduce human contact and interaction. Accordingly, the majority of respondents assumed that older adults would feel more isolated by using care robots [40].

### Influence of Care Scenario Intimacy on Affective Attitudes

The existing literature further suggests that people discriminate in their acceptance of robots for different care tasks. The use of robots to perform daily routine activities in nursing homes has garnered approval, as this could reduce the workload and physical burden of human caregivers [22, 30, 52]. Moreover, robots that provide services for care recipients, such as picking up and carrying things [23, 30, 31], or bringing drinks and food [30, 32], are also accepted. Study participants also perceived the management of emergency situations (such as placing calls for help in the event of a fall) as a conceivable task for assistance robots. The same was true for reminder and monitoring functions (e.g., issuing medication reminders or measuring blood pressure) [23, 31, 33].

In contrast, study participants have been unenthusiastic about assigning robots tasks which would involve social interaction or physical contact. For example, in studies by Lehmann et al. [23] and Smarr et al. [24], social interaction (e.g., having a conversation) with a robot was hardly imaginable for most participants. Saplacan et al. [52] also pointed out that actively encouraging older people to interact with care robots as if they were companions may make them feel deceived and infantilized. However, nursing and medical students participating in a study by Łukasik et al. [33] considered facilitative social functions, such as encouraging contact with friends, to be suitable for a care robot, as this could help elderly people feel less lonely and thus improve their mood. Robot assistance for nursing activities, such as help with personal hygiene, is usually rejected [22–24]. Patient bathing is a significant part of nursing care, because washing the body is one of the most complex activities of daily living and, thus, among the first that elderly people can no longer perform independently [55]. However, receiving help with personal hygiene is also a very intimate process and is often perceived as shameful, as it involves direct physical contact with another person and draws their attention to intimate bodily functions and possibly the inability to control them [18, 22]. Substituting the “human element of care” with assistive technology could therefore potentially reduce feelings of shame among care recipients [18]. Consistent with this, there are indications that some people could imagine or even prefer using a robot for personal care [20, 22]. However, as Klein et al. [22] point out, personal care activities demand particularly high level of trust between caregiver and care recipient. Based on the above literature, it is questionable whether care robots can elicit this level of trust in humans.

### Influence of Temporal Distance on Affective Attitudes

In our study, we used text vignettes to ask participants to imagine themselves in two different nursing care scenarios. This method requires participants to think beyond their current life circumstances and imagine potentially counterfactual situations [44]. Yet, the development of care robots is still in its infancy, and the technology is neither fully mature nor widely available. Therefore, it could be argued that the idea of being cared for by a robot *today* might seem overly futuristic to our participants. Consequently, participants might be more willing to engage with the idea of being cared for by robots in the more distant future. Confrontation with scenarios that are perceived as implausible in the present may jeopardize participants’ focus on the vignette dimensions causing the implausibility and, thus, negatively affect data validity [56]. This would recommend placing the hypothetical care scenarios in the more distant future.

We conducted our study online using a US convenience sample from CloudResearch’s Prime Panels. Although the diversity of participants in online experiments has increased, the majority are still younger than the US average and are largely younger than 60 years old [57]. The need for long-term care usually occurs at an older age. Therefore, we anticipated that long-term care dependency might be a purely hypothetical situation for most of our participants, since most probably did not expect to face care dependency until the very distant future. An intrapersonal empathy gap leads people to project their current emotional state onto the future and thus underestimate how their views might change [58]. As a result, it may be very difficult for them to anticipate how they would feel in future care scenarios. Too great a difference between the characters in a vignette and the study’s participants could be problematic, as others have noted (see, e.g., 41), and potentially skew the results. Therefore, to enhance participants’ ability to put themselves in the described care scenarios, we sought to include care scenarios that occur in the near future.

However, the temporal distance from an event (i.e., the time span that lies between a person's present and that event) influences how it is perceived. For example, if an action lies in the more distant future, arguments in favor of that action seem to be more salient [59]. People, therefore, tend to have more positive attitudes toward this action than if the same action lies in the nearer future [60]. For this reason, we decided to control for possible biases in attitudes caused by the participants’ temporal distance from the care scenarios by comparing their reactions to vignettes set in a hypothetical present with their reactions to vignettes set in a hypothetical future.

### Influence of Experience with Care Dependency on Affective Attitudes Toward Care Robots

Studies on the general acceptance of robots have found that older people have less positive attitudes toward robots than younger people do, as do women compared to men [38, 40, 61]. Social interaction with a robot, especially if this would be its main function, is often rejected by elderly people [53]. Similarly, older people are mostly opposed to the use of robots for tasks related to personal care or leisure activities [24, 31]. However, in their systematic literature review, Savela et al. [35] found that older people had more positive attitudes toward assistive robots in elderly care than professional caregivers did and were more likely to have positive attitudes overall than negative attitudes [35]. Surprisingly, Smarr et al. [24] found that the older participants in their study even preferred robot assistance over human assistance for service tasks such as doing household chores, moving items, providing news, or reminding people of their appointments. The elderly participants in an experimental study by Beedholm et al. [62] did not categorically reject the use of a robotic bathtub with human assistance, but also did not find the tested application useful. In contrast, in a study by Pino et al. [63], individuals with mild cognitive limitations rated care robots as more useful than healthy elderly participants did and showed greater intention to use them. This is consistent with the work of Honekamp et al. [30], who assumed that many elderly people already have support needs that could be met by new assistive technologies, which is why they see a concrete benefit in their use and are more positive about them. In contrast, Hoppe et al. [51] found that participants who rated their health as "not good" (as opposed to participants with "good" health status) preferred a human caregiver to a care robot. It seems conceivable that an individual’s actual need for care changes their appreciation of specific technologies that provide support under their living conditions. That care-dependent people may have different attitudes toward assistive technologies than people who are not care-dependent have may be explained by an interpersonal empathy gap. This gap makes it difficult for people to imagine that people’s preferences in very different emotional situations may differ from their own [64]. However, study findings on (potentially) care-dependent people's views toward care robots are currently inconclusive (e.g., 9). At present, therefore, it is not clear whether this divergence in preferences leads to more positive or negative attitudes toward care robots among people in need of care.

## Methods

### Hypotheses

#### Attitude Toward Care Robots Compared to Human Caregivers

Given the empirical evidence of and ethical deliberations on general attitudes toward care robots, we assumed that, in direct comparison with human caregivers, care robots would be perceived less positively than their human counterparts. This led to our first hypothesis:

##### H1

People’s perceived comfort level with care robots is lower than with human caregivers.

#### Influence of the Scenario’s Intimacy on Attitudes Toward Caregivers

Taking our hypothesis on people’s general attitudes toward care robots (H1) and the findings on the perception of robots in different care scenarios cited above, we expected that an intimate care scenario would induce a stronger negative impact on a person’s affective attitude toward a care robot than a non-intimate care scenario would. Accordingly, we formulated our second hypothesis:

##### H2

The gap between people’s perceived comfort levels with care robots and human caregivers is larger in an intimate care scenario than in a non-intimate care scenario.

#### Influence of Temporal Distance on Attitudes Toward Caregivers

As described above, in the context of a vignette study on care robots, there are certain advantages to asking participants to imagine themselves in a scenario with a care robot in the near and others to proposing a similar scenario in the more distant future. Since it is unclear to what extent temporal proximity’s effect dominates that of the maturity of care robots (or the inverse), we did not formulate a directional hypothesis about the influence of a hypothetical situation’s temporal distance on affective attitudes toward different caregivers. Therefore, our third hypothesis is:

##### H3

The gap between people’s level of comfort with care robots and with human caregivers depends on temporal distance from the care scenarios.

#### Influence of Experience with Care Dependency on Affective Attitudes Toward Caregivers

Studies of differing attitudes toward care robots according to differing experiences of needing nursing care are currently inconclusive. Therefore, we could only formulate a nondirectional hypothesis about the influence of care dependency experience on affective attitudes toward different caregivers:

##### H4

The gap between people’s perceived comfort level with care robots and human caregivers depends on whether people are affected by care dependency or not.

### Study Design

For this study, we used a text vignette methodology to evaluate and compare people’s attitudes toward care robots and those toward human caregivers. We introduced participants to the vignettes by asking them to put themselves in the situation of unexpectedly needing nursing care and having to move to a long-term care facility (see Appendix A in Supplementary Information for the complete wording of the vignettes). The introductory text explained that in this care facility, human caregivers and care robots share the work. Participants were randomly assigned to one of four experimental conditions (see 3.3 and Table 1), so that approximately half of the participants read that this situation would occur tomorrow, and the other half read that it would occur in 25 years. About half of each group read that a human caregiver was responsible for their ward, while the other half read that they would live in a ward managed by a care robot. We emphasized our particular interest in their attitude toward the caregiver responsible for them, intending to bring the nature of the caregiver into the participants’ focus for the subsequent questions. Participants then read two vignettes featuring the same two characters: a caregiver and a care recipient (the participant). One vignette described an intimate care scenario of the care recipient being helped with personal hygiene by the caregiver (*intimate scenario*). The other vignette dealt with a non-intimate service-oriented scenario in which the care recipient received a glass of water and was nudged by the caregiver to drink something (*non-intimate scenario*). Because we focused on participants’ affective reactions to the two different caregivers in general, rather than on their perceptions of specific characteristics as positive or negative, the vignettes did not include information or illustrations about the specific appearance or characteristics of the human caregiver and the care robot.

**Table 1:** Overview of experimental design

	Between subjects			
	Human × tomorrow	Human × in 25 years	Robot × tomorrow	Robot × in 25 years
Within subjects*	Non-intimate	Non-intimate	Non-intimate	Non-intimate
	Intimate	Intimate	Intimate	Intimate
Condition	H0	H25	R0	R25

*Non-intimate and intimate scenarios were shown in randomized order

### Vignette Design

The experiment used a 2 × 2 × 2 mixed-design and text vignettes, featuring two between-subjects manipulations and one within-subjects manipulation. For the between-subjects manipulations, we chose the nature of the caregiver and the temporal distance, according to which the vignettes described either a *human* or a *robot* caregiver and that the care scenarios would take place either *tomorrow* or *in 25 years*.

We varied the nature of the caregiver—*human* versus *robot—*to test whether and how people’s affective attitudes differ between caregivers. To increase participants’ ability to engage with the described care scenarios (temporal proximity) on the one hand, and to mitigate possible biases due to perceived implausibility (maturity of care robot technology) on the other, we further varied the temporal distance, so that half of our vignettes described care scenarios taking place *tomorrow*, while the other half described these situations as taking place *in 25 years*. This resulted in four experimental conditions to which participants were randomly assigned: *human* × *tomorrow* (henceforth “H0”), *human* × *in 25 years* (“H25”), *robot* × *tomorrow* (“R0”), and *robot* × *in 25 years* (“R25”, see Table 1).

We designed the intimacy of the care scenario as a within-subjects manipulation and presented each participant with two scenarios: an *intimate scenario* (help with personal hygiene) and a *non-intimate scenario* (getting something to drink). To address issues of ordering, we balanced the study so that the two scenarios were shown in a randomized order.

### Measures

#### Perceived Level of Comfort

We assessed participants’ affective attitude toward caregivers on the basis of their self-reported level of comfort in the described care scenarios. For each of the two vignettes, participants rated the statement “I feel comfortable in the described situation” on a 7-point Likert scale from 0 (*completely disagree*) to 6 (*completely agree*).

#### Demographic Factors

As outlined in Sect. 2, age and gender have been found to influence people’s general attitudes toward robots [38, 40, 61]. However, some studies have suggested that older people—thus, potentially, actual care recipients—evaluate care robots more positively than, for example, caregivers do [35]. For these reasons, we asked participants to indicate not only their age and gender but also their care dependency status. The latter was determined via a self-assessment with the closed question “Are you in need of nursing care?”.

#### Attitudes Toward Robots

To control for the potential influence of participants’ general attitudes toward robots on their comfort ratings, we included the English version of the Negative Attitudes toward Robots Scale (NARS) by Nomura et al. [65] in the post-experimental questionnaire. This 14-item self-report inventory is the most widely used psychometrically validated scale for assessing the social acceptability of robots [66]. The NARS consists of three subscales, of which two are considered to capture affective attitudes and one to measure cognitive attitudes toward robots [34]. The first subscale, Negative Attitudes toward Situations of Interaction with Robots, captures affective attitudes (hereafter referred to as the NARS.Interaction). An example item reads: “I would feel nervous operating a robot in front of other people”. The second subscale, Negative Attitudes toward the Social Influence of Robots, targets cognitive attitudes (NARS.Influence; e.g., “I am concerned that robots would be a bad influence on children”). The third subscale, Negative Attitudes toward Emotions in Interactions with Robots, again captures affective attitudes (NARS.Emotions; e.g., “I would feel relaxed talking with robots”). Participants rated each item on a 5-point Likert scale, with anchors of 1 (*strongly disagree)* and 5 (*strongly agree)*. Three items on the scale are positively worded; for these, the scores are reversed, so that for all items higher scores reflect more negative attitudes (see questionnaire in Appendix A in Supplementary Information).

### Participants and Procedure

The experiment was conducted online in June 2020 using the survey tool SoSci Survey [67]. Participants were volunteers recruited from the CloudResearch platform’s Prime Panels [57]. Online research platforms such as CloudResearch have been widely used in the social sciences, as they have been shown to be a reliable and valid source of experimental data across a variety of tasks and countries [68–71]. In terms of age, family background, religiosity, education, and political views, Prime Panel participants are more diverse and more representative of the US population than, for example, MTurk participants or traditional university subject pools [57]. Using SoSci Survey, we randomly assigned participants to one of the four conditions (H0, H25, R0, or R25, see Table 1). Multiple participation was precluded by CloudResearch. After an introduction, the two care scenario vignettes were presented to the participants one by one, and they rated their perceived level of comfort for each situation. After the experimental task, the participants’ attitudes toward robots were assessed. The last step asked the participants to complete a questionnaire that covered the three demographic items listed in 3.4 (regarding gender, age, and care dependency status). In addition, the participants responded to a list of supplemental questions for exploratory purposes not related to this study. Out of 1413 people who opened the questionnaire, 140 (9.9%) failed to complete it. The remaining 1273 participants were included in our analysis. They ranged in age from 18 to 92 years (*M* = 47.3, *SD* = 18.2). As only very few participants reported their gender as non-binary, we limited our analysis to the binary gender categories. Of the 114 participants who reported needing nursing care at the time of the experiment, 73.7% (84) were male. Table 2 depicts the sample’s characteristics.

**Table 2:** Socio-demographic characteristics

Baseline characteristic	H0		H25		R0		R25		Full sample	
	*n*	%	*n*	%	*n*	%	*n*	%	*n*	%
Gender										
Female	169	57.3	213	62.6	181	58.4	193	58.8	756	59.4
Male	122	41.4	123	36.2	129	41.6	131	39.9	505	39.7
In need of nursing care ^a^	25	8.5	32	9.4	29	9.4	28	8.5	114	9.0

	*M *(*SD*)	*M *(*SD*)	*M *(*SD*)	*M *(*SD*)	*M *(*SD*)
Age	45.7 (18.3)	48.3 (18.6)	47.3 (18.1)	47.5 (18.0)	47.3 (18.2)

*N* = 1273. No statistically significant differences were found between conditions in age (all *p* ≥ 0.08, unpaired *t*-tests, two-sided), gender (all *p* ≥ 0.20, χ^2^ tests), and care-dependency (all *p* ≥ 0.78,  χ^2^ tests). Nine participants did not report their age, and 12 participants reported no or non-binary gender

^a^Reflects the number and percentage of participants answering “yes” to this question

A total of 635 participants read the vignettes involving a *human caregiver* and 638 read the ones involving a *care robot*. Of these, 340 and 328 participants, respectively, were assigned to the temporal distance *in 25 years*, while the remaining participants were assigned to the temporal distance *tomorrow*. Each participant encountered two care scenarios: one describing an intimate scenario and one describing a non-intimate scenario (see Table 1).

### Ethical Considerations

The investigation was conducted according to the principles expressed in the Declaration of Helsinki. Informed consent was obtained from the participants via the survey platform. Overall, the study took about 20 minutes and participants were compensated with a fixed amount of $1.75 for completion. They could withdraw from the study at any time without consequences for them. The participants could skip any questions in the survey that they did not want to answer. The data of the experiment was only stored locally, on the computers of the researchers.

## Results

In the first step of our analysis, we investigated the influence of the nature of the caregiver (human vs. robot; H1) on comfort levels. Second, we evaluated the interactive influence of caregiver and scenario (H2) and of caregiver, scenario, and temporal distance (H3) on perceived comfort. Third, we examined whether and how experience with care dependency influences comfort levels (H4). In all steps of analysis, we used two-sided non-parametric Wilcoxon rank-sum tests for the independent conditions (i.e., the effects of caregiver nature and temporal distance from the onset of care dependency) and Wilcoxon signed-rank tests for the dependent conditions (i.e., those including the scenarios). Following the non-parametric analysis, we conducted regression analyses to assess the robustness of our results. We also included participants’ age, gender, and attitudes toward robots as additional covariates in our models.

### Main Effects

**Fig. 1:**
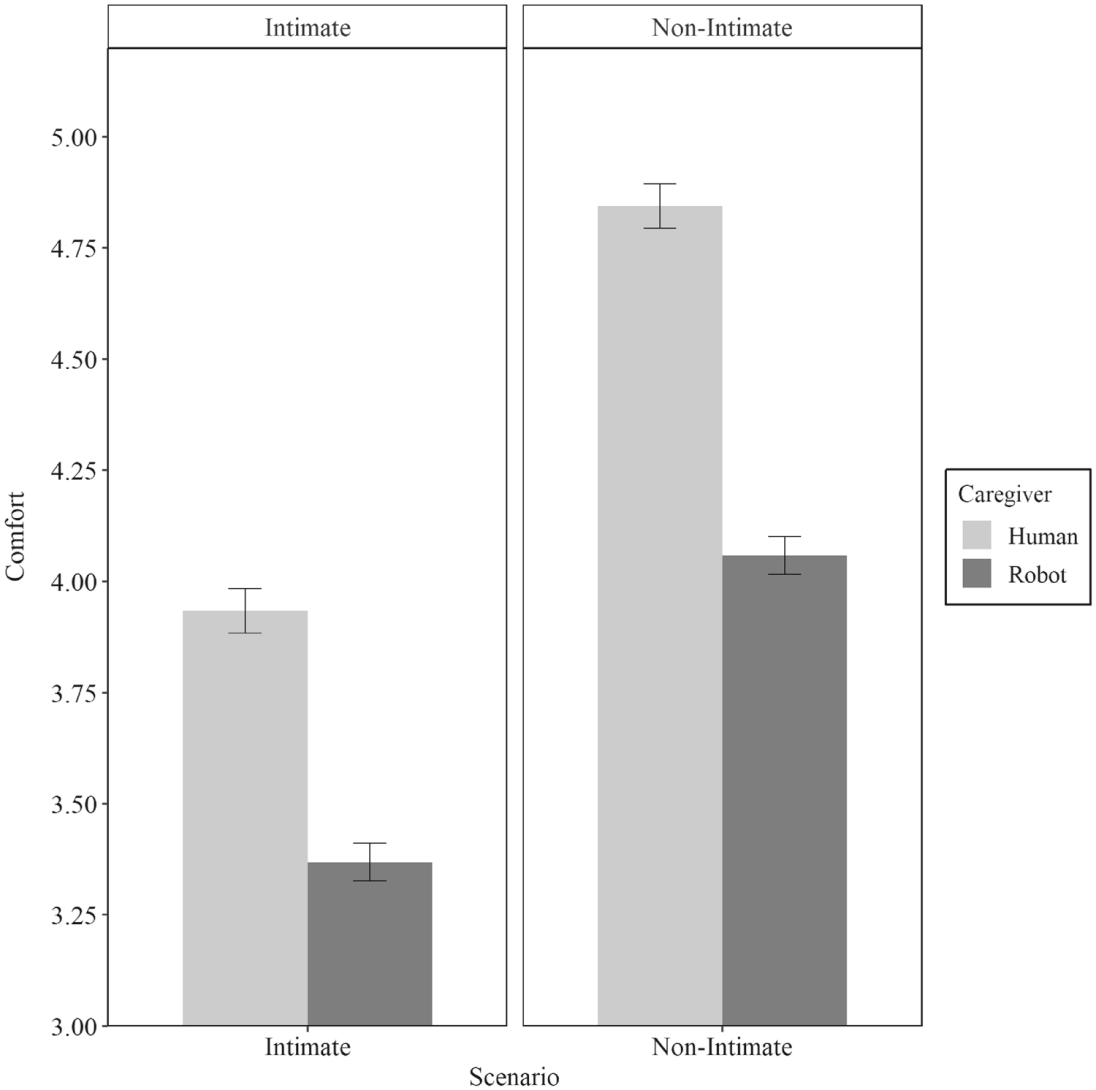
Perceived comfort, depending on caregiver nature and scenario intimacy

On average, participants reported feeling comfortable in the care scenarios (*M* = 4.05, *SD* = 1.54). The effects of the experimental manipulation on participants’ comfort can be inferred from Fig. 1.

#### Nature of the Caregiver

We started with the hypothesis that people’s affective attitudes toward care robots would be more negative than their affective attitudes toward human caregivers. As predicted, participants in the care robot conditions expressed a significantly lower comfort level (*M* = 3.71, *SD* = 1.72) than in the human caregiver conditions (*M* = 4.39, *SD* = 1.25; *p* < 0.001, *d* = 0.45). This supports Hypothesis H1, that people would prefer being cared for by humans than by robots.

#### Intimacy of the Scenario

We also expected that negative affective attitudes toward care robots would be more pronounced in intimate care scenarios than in non-intimate care scenarios (H2). First, we found that the order in which the scenarios were presented had no impact on participants’ comfort ratings. Therefore, we merged the data from the two orders in our analyses. Comparing the two scenarios revealed participants’ comfort level to be significantly higher in the non-intimate scenario (*M* = 4.45, *SD* = 1.16) than it was in the intimate scenario (*M* = 3.65, *SD* = 1.16; *p* < 0.001, *d* = − 0.46).

Concerning the interactive influence of caregiver and scenario, we found that in the intimate scenario, participants imagining themselves with a human caregiver reported comfort levels that were significantly higher (*M* = 3.93, *SD* = 1.25) than those reported by the participants imagining the care robot scenario (*M* = 3.37, *SD* = 1.06; * p* < 0.001, *d* = 0.31). The same was true for participants in the non-intimate scenario with a human caregiver (*M* = 4.84, *SD* = 1.25) who reported higher comfort levels than participants in the same scenario, but with a care robot (*M* = 4.06, *SD* = 1.06; *p* < 0.001, *d* = 0.50). Figure 1 summarizes our findings. However, the difference between reported comfort levels for human and robot caregivers was smaller in the intimate scenario (0.56) than in the non-intimate scenario (0.78), refuting H2. Thus, contrary to our hypothesis, participants’ robot aversion was stronger in the non-intimate scenario than in the intimate scenario.

#### Temporal Distance

We further expected that the care scenarios’ description as occurring either tomorrow or in the more distant future would influence affective attitudes. In contrast to this assumption, the time of onset of the need for nursing care (temporal distance) did not significantly affect participants’ comfort level. Temporal distance affected participants’ comfort levels significantly neither in interaction with the caregiver, nor in interaction with both the caregiver and the scenario (see Table B.1 in Appendix B in Supplementary Information for the respective *p*-values). Hypothesis H3 was thus refuted. For the analyses that followed, therefore, we stopped distinguishing between the temporal distances *tomorrow* and *in 25 years*.

#### Experience with Care Dependency

**Fig. 2:**
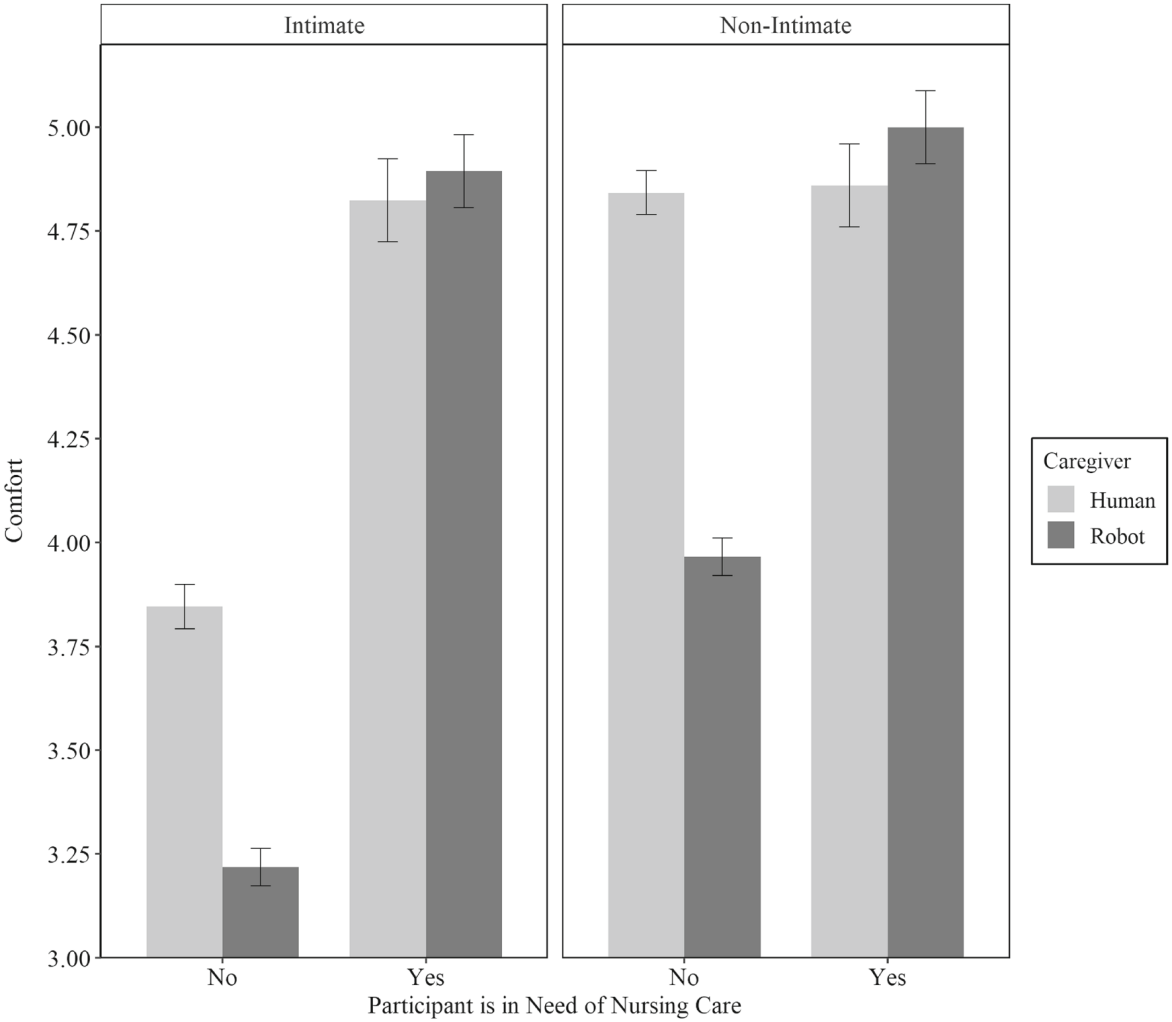
Participants’ perceived comfort depending on need for nursing care, caregiver nature, and scenario intimacy

Last, we expected that being affected by care dependency would influence the gap between people’s perceived comfort level with care robots and that with human caregivers. Figure 2 reveals a remarkably positive influence of participants’ own experience with care dependency on perceived comfort in both scenarios. Participants who identified themselves as in need of nursing care reported significantly higher comfort levels than did non-care-dependent participants, in general (i.e., regardless of caregiver nature or scenario intimacy); both with a human caregiver and with a care robot (i.e., regardless of the scenario intimacy); with a care robot in both scenarios; and with a human caregiver in the intimate scenario. Numeric values (means and corresponding *p*-values of non-parametric tests) can be inferred from Table B.2 in Appendix B in Supplementary Information. Furthermore, we found that in both scenarios care-dependent participants’ comfort levels did not differ significantly with caregiver nature. By contrast, non-care-dependent participants rated their level of comfort with a care robot significantly lower than their level of comfort with a human caregiver in both scenarios. Hypothesis H4 was thus supported.

### Robustness Checks

Subsequently, we conducted a series of multiple regression analyses with the comfort level as the dependent variable. In doing so, we aimed to test the robustness of our non-parametric findings when controlling for other demographic characteristics (age and gender) and participants’ attitudes toward robots. For the further analysis, the dichotomous variables were dummy-coded as specified in Table 3.

**Table 3:** Designation of dummy-coded, dichotomous independent variables

Variable	Category (Variable value = 1)	Reference category (Variable value = 0)
Caregiver	Robot	Human
Scenario	Intimate	Non-intimate
Care dependency status	Care-dependent	Non-care-dependent
Gender	Female	Male

We started by replicating our non-parametric findings concerning the main effects of the caregiver and scenario on comfort. Consistent with our previous results, the caregiver nature (*Robot*, see Model 1 in Table 4) and the scenario intimacy (*Intimate scenario*, see Model 2) were significant predictors of comfort. Model 3 further shows a significant interaction effect of the caregiver and the scenario (*Robot* × *intimate scenario*). This indicates, first, that the specific nature of the caregiver influenced participants’ comfort differently in the two scenarios and, second, that this influence was less negative in the intimate scenario. Taken together, these results reinforce our previous findings regarding H1 and H2, namely, that people value care robots below their human counterparts; however, these more negative affective attitudes are *not* more pronounced in an intimate scenario than in a non-intimate scenario.

**Table 4:** Regression results for experimental manipulation

Independent variable	Dependent variable: Comfort		
	(1)	(2)	(3)
Robot	− 0.676^***^		− 0.786^***^
	(0.084)		(0.088)
Intimate scenario		− 0.800^***^	− 0.910^***^
		(0.046)	(0.070)
Robot × intimate scenario			0.221^**^
			(0.092)
Constant	4.389^***^	4.450^***^	4.844^***^
	(0.045)	(0.045)	(0.052)
Observations	2546	2546	2546
Participants	1273	1273	1273
Adjusted R^2^	0.035	0.050	0.085

Robust standard errors, clustered at the individual subject level, are shown in parentheses. In all models, the dependent variable is the degree of comfort subjects claimed to feel in the situation described. Each subject evaluated two care scenarios: an intimate scenario (help with personal hygiene) and a non-intimate scenario (getting something to drink)

**p < 0.05; ***p < 0.01

Subsequently, we tested the influence of care dependency on participants’ perceived comfort and controlled for the possible influence of age and gender. Therefore, in a second step, we split the dataset by scenario (see Table 5). This confirmed, first, the care robot’s significant negative effect on perceived comfort in both scenarios (see Table 5, *Robot* in all models). Including participants’ care dependency status in the regression analyses showed, second, a strong positive influence of care dependency on comfort levels in both scenarios (see Table 5, *Care-dependent* and *Robot* × *care-dependent* in all models); this finding aligns with our non-parametric test results. For the intimate scenario, regression coefficients indicated that care-dependent participants generally felt significantly more comfortable than did participants who had not experienced the need for nursing care, both with a human caregiver and with a care robot. In the non-intimate scenario, there was no general difference between care-dependent and non-care-dependent participants’ comfort level with the human caregiver. However, with a care robot, care-dependent participants felt significantly more comfortable than non-care-dependent participants did. Overall, these results support Hypothesis H4: needing nursing care influences how people rate their comfort level with care robots in comparison to that with human caregivers.

**Table 5:** Influence of caregiver and socio-demographic factors on comfort

Independent variable	Dependent variable: Comfort					
	Intimate scenario			Non-intimate scenario		
	(1)	(2)	(3)	(4)	(5)	(6)
Robot	− 0.632^***^	− 0.637^***^	− 0.598^***^	− 0.887^***^	− 0.887^***^	− 0.638^***^
	(0.109)	(0.108)	(0.168)	(0.093)	(0.093)	(0.148)
Care-dependent	0.981^***^	0.749^***^	0.870^***^	0.013	0.038	0.170
	(0.198)	(0.204)	(0.207)	(0.181)	(0.188)	(0.194)
Robot × care-dependent	0.680^**^	0.682^**^	0.459	1.009^***^	1.005^***^	0.747^**^
	(0.285)	(0.283)	(0.298)	(0.279)	(0.278)	(0.291)
Age		− 0.146^***^	0.038		0.043	0.145^***^
		(0.051)	(0.066)		(0.043)	(0.050)
Female		− 0.395^***^	− 0.375^***^		− 0.004	0.188
		(0.106)	(0.143)		(0.092)	(0.115)
Robot × age			− 0.373^***^			− 0.206^**^
			(0.101)			(0.087)
Robot × female			− 0.032			− 0.375^**^
			(0.211)			(0.184)
Constant	3.843^***^	4.104^***^	4.081^***^	4.847^***^	4.847^***^	4.719^***^
	(0.073)	(0.099)	(0.116)	(0.055)	(0.084)	(0.097)
Observations	1256	1256	1256	1256	1256	1256
Adjusted R^2^	0.065	0.081	0.089	0.074	0.074	0.080

Robust standard errors are shown in parentheses. The metric variable *age* was standardized before performing the regression analysis

^**^p < 0.05; ***p < 0.01

The inclusion of age and gender in the regression analyses revealed that these factors had varying influence on comfort levels across scenarios and caregivers but did not mitigate the impact of care dependency (see Table 5).

#### Influence of Attitudes Toward Robots

We evaluated the robustness of our findings for the care robot condition by controlling for participants’ robot aversion. Using the participants’ responses to the Negative Attitudes toward Robots Scale (NARS) [65], we first performed a reliability analysis of the three subscales using Cronbach’s alpha. We removed one item from each of the NARS.Interaction and NARS.Influence scales due to low item-total-correlation.^3^ Due to the very small number of missing answers for the other items, these were imputed with the corresponding item mean.^4^ The revised scales showed good internal consistency, with Cronbach’s alphas of α = 0.85 for NARS.Interaction, α = 0.76 for NARS.Influence and α = 0.80 for NARS.Emotions. The subscale scores were then calculated as sums of the corresponding items as suggested by Nomura, Kanda, Suzuki [75].

High scores on the NARS.Interaction and NARS.Emotions scales—i.e., strongly negative attitudes toward situations of interaction with robots and toward emotions in interactions with robots, respectively—significantly reduced participants’ perceived comfort with a care robot in both scenarios (see Table 6, *NARS.Interaction* and *NARS.Emotions* in Models 2 and 4). However, participants’ scores on the NARS.Influence scale (i.e., their levels of negative attitudes toward the social influence of robots) did not have a significant effect (see Table 6, *NARS.Influence* in Models 2 and 4). Controlling for participants’ attitudes toward robots almost completely mitigated the influence of age and gender on perceived comfort with care robots (see Table 6, *Age* and *Female* in Models 2 and 4). Although controlling for attitudes toward robots also mitigated the impact of care dependency on perceived comfort, care dependency still significantly influenced participants’ comfort ratings with care-dependent participants feeling more comfortable in both scenarios (see Table 6, *Care-dependent* in Models 2 and 4).

**Table 6:** Influence of age, gender, care dependency, and NARS scores on perceived comfort with a care robot

Independent variable	Dependent variable: Comfort			
	Intimate scenario		Non-intimate scenario	
	(1)	(2)	(3)	(4)
Care-dependent	1.329^***^	0.736^***^	0.917^***^	0.475^**^
	(0.214)	(0.209)	(0.218)	(0.219)
Age	− 0.331^***^	− 0.243^***^	− 0.060	0.004
	(0.075)	(0.063)	(0.070)	(0.062)
Female	− 0.406^***^	− 0.052	− 0.188	0.108
	(0.154)	(0.137)	(0.144)	(0.129)
NARS.Interaction		− 0.364^***^		− 0.363^***^
		(0.112)		(0.104)
NARS.Emotions		− 0.966^***^		− 0.788^***^
		(0.077)		(0.080)
NARS.Influence		0.007		− 0.003
		(0.112)		(0.108)
Constant	3.482^***^	3.324^***^	4.080^***^	3.944^***^
	(0.121)	(0.106)	(0.112)	(0.101)
Observations	631	631	631	631
Adjusted R^2^	0.095	0.380	0.025	0.270

Robust standard errors are shown in parentheses. Metric variables *age*, *NARS.Interaction, NARS.Emotions* and *NARS.Influence* were standardized before the regression analysis was performed

^**^p < 0.05; ***p < 0.01

Finally, to further examine the differences between participants who reported needing nursing care and those who did not, we divided the dataset according to people’s care dependency status (see Table 7). This division revealed differences in the influence of age, NARS.Interaction and NARS.Emotions on perceived comfort: In the intimate scenario, older non-care-dependent participants felt significantly less comfortable than younger participants did (see Table 7, *Age* in Model 2), whereas the comfort of care-dependent participants was not affected by their age (see Table 7, *Age* in Model 1). In contrast, in the non-intimate scenario, older care-dependent participants felt significantly more comfortable than younger participants did (see Table 7, *Age* in Model 3), while there was no difference among non-care-dependent participants (see Table 7, *Age* in Model 4). NARS.Interaction scores significantly influenced non-care-dependent participants’ comfort levels, with stronger negative attitudes leading to lower perceived comfort (see Table 7, *NARS.Interaction* in Models 2 and 4). NARS.Emotions scores had a significant negative effect on all participants’ comfort levels in both scenarios. However, larger regression coefficients for the care-dependent participants suggest that these participants’ negative attitudes toward emotions in interactions with robots had a stronger negative impact on their perceived comfort than the same attitudes of their non-care-dependent counterparts did on theirs (see Table 7, *NARS.Emotions* in all models).

**Table 7:** Regression results: Care-dependency split for care-robot condition

Independent variable	Dependent variable: Comfort			
	Intimate scenario		Non-intimate scenario	
	Care- dependent	Non-care- dependent	Care- dependent	Non-care- dependent
	(1)	(2)	(3)	(4)
Age	0.337	− 0.271^***^	0.605^**^	− 0.028
	(0.264)	(0.065)	(0.294)	(0.064)
Female	0.092	− 0.022	0.007	0.159
	(0.508)	(0.142)	(0.569)	(0.134)
NARS.Interaction	0.272	− 0.423^***^	0.148	− 0.425^***^
	(0.224)	(0.119)	(0.304)	(0.110)
NARS.Emotions	− 1.033^***^	− 0.935^***^	− 1.015^***^	− 0.740^***^
	(0.228)	(0.082)	(0.282)	(0.086)
NARS.Influence	− 0.188	− 0.003	− 0.017	− 0.030
	(0.186)	(0.120)	(0.249)	(0.116)
Constant	3.926^***^	3.299^***^	4.229^***^	3.905^***^
	(0.372)	(0.108)	(0.451)	(0.104)
Observations	55	576	55	576
Adjusted R^2^	0.339	0.351	0.300	0.261

Robust standard errors are shown in parentheses. Metric variables a*ge, NARS.Interaction, NARS.Emotions* and *NARS.Influence* were standardized before the regression analysis was performed

**p < 0.05; ***p < 0.01

## Discussion

In the not-too-distant future, many societies will face a large-scale shortage of specialized caregivers due to an aging population and the decreasing appeal of the nursing profession. One strategy for meeting the challenges of the growing gap between the demand and supply of professional caregivers is the use of assistive technologies and robots to supplement human caregiving. In current public discourse, the development of robots that can perform caregiving tasks is often rejected as inhumane and inappropriate. The opinion leaders in this discourse mostly do not (yet) depend on the support of others. However, as other fields’ studies involving vulnerable groups have shown, discourse about a technology’s ethical and social acceptability must include the perspective of those affected by its use, as their evaluative criteria may differ significantly from those of the non-affected (e.g., likeability and positive affect, rather than the concepts of dignity or deception) [14, 76, 77].

In this paper, we provided insights into how people perceive the use of care robots, while controlling for care dependency’s effect on this perception. We found support for the notion that people prefer human caregivers to robot caregivers. However, while participants who are not (yet) in need of care strongly devalued care robots compared to human caregivers, care-dependent participants did not express such a devaluation. Instead, they did not differentiate between the caregivers’ nature and the care scenarios in reporting their perceived comfort. The fact that the care-dependent participants generally expressed more positive affective attitudes toward both caregivers than the non-care-dependent participants did suggests that their similar evaluation of human and robot caregivers stems not from a more misanthropic attitude but from a lower aversion to robots. The results proved robust when respondents’ general attitudes toward robots were considered.

Contrary to our assumption and the findings of previous studies (see for instance [30] and [24]), robot aversion was found to be stronger in the non-intimate scenario than in the intimate scenario. A possible explanation for this finding could be that the idea of receiving help with personal hygiene is generally rather unpleasant for many people [18, 20], especially if they lack personal experience of needing nursing care, so a caregiver’s specific nature has less influence on a person’s comfort than it might in a more physically distanced, service-oriented care scenario. Artificial caregivers’ literal de*human*ization and de*person*alization of such a potentially shame-inducing situation could well be perceived as positive for one’s privacy, independence, and dignity [18, 20]. Therefore, the idea of a non-judgmental, neutral robot providing care in an intimate situation instead of a human caregiver doing so could mitigate people’s aversion to robots to some extent. Furthermore, previous studies have suggested that, while the use of robots to provide services such as fetching drinks or food is widely accepted [30, 32], social interactions (such as conversations) with robots are generally not valued [23, 24]. In addition to the core activities of *getting help with personal hygiene* and *getting a glass of water*, both of our scenarios included conversations with the caregiver. This may have drawn more focus from participants in the non-intimate scenario than it did in the intimate scenario, and thus may have had a greater impact on their affective attitudes. Negative emotions elicited by the interaction described between the care robot and the care recipient, therefore, may have outweighed participants’ possible positive or neutral emotions toward the “service” part of the care scenario. Further research is needed to disentangle these effects.

Our sample’s insensitivity to the temporal distance from the onset of care dependency could stem from an absent affinity for or experience with robotics. Presumably, the participants were unfamiliar with many of the current and possible future developments in the field of robotics. People generally orient themselves toward familiar and established concepts and everyday experiences, or as Carbon puts it: “If you ask people about the future, they will talk about the world of today” [78, p. 6]. In our text-based vignette study, it might therefore have been (too) challenging for participants to imagine care robots’ possible future state of development, and, thus, they tried “to extrapolate the present time with some ingredients of the latest innovations” [78, p. 6] in their assessments. This may have led to the absence of significant differences between participants’ assessments of their subjective level of comfort in the two temporal conditions.

Consistent with previous studies [38, 40, 61], our results suggest prima facie that women are less comfortable with care robots than men are. Therefore, because our study included more female than male participants, we may have a particularly robot-critical sample. This concern is somewhat mitigated by a closer look at the data. When we controlled for participants' attitudes toward robots in general, the gender differences were completely mediated. This is consistent with Flandorfer’s systematic literature review, which found that attitudes toward robots can potentially mediate the influence of sociodemographic factors on the acceptance of care robots [79]. Nevertheless, future studies should challenge our results with a more balanced sample to disentangle possible gender effects.

Controlling for participants’ attitudes toward robots also almost completely mediated the influence of age on participants’ comfort with care robots. Moreover, strongly negative attitudes toward interactive situations with robots led to more negative affective attitudes toward care robots among non-care-dependent participants, but not among current care recipients. Two explanations seem plausible here: First, those currently receiving care may already be more familiar with the use of other assistance technologies and, therefore, also see a greater benefit in the use of care robots than people who do not need assistance (see also 30). Second, care recipients are certainly more familiar with human caregivers. They may have experienced certain limitations of human caregiving (such as that even human caregivers cannot provide unlimited support in daily life) and, therefore, do not devalue care robots in comparison to human caregivers as much as the non-care-dependent do. Unlike the latter, they are able to compare a potential future technology to the actual state of caregiving rather than an ideal image of caregiving. This could influence care recipients’ affective and cognitive attitudes towards care robots, about which they might feel more positive, but which they also might see as having certain advantages (e.g., in terms of privacy or greater autonomy) over human caregiving. As a result, current care recipients’ affective attitudes toward care robots in particular might be less influenced by their general attitude toward interaction with robots.

Finally, participants’ attitudes toward emotions in interactions with robots strongly influenced their affective attitudes toward care robots: the intimate care scenario in our study, in particular, is a situation in which people often feel vulnerable, and thus “want to feel respected and cared for” [80, p. 1]. Our results suggest that, in scenarios with care robots, the participants who could imagine robots as able to express emotions such as compassion and who would perceive this ability positively (i.e., those with low scores on the NARS.Emotions scale), felt significantly more comfortable than those who could not. This implies, first, that even though care robots may not be able to provide “genuine” (in the sense of “human”) care, they do not necessarily diminish people’s perceived comfort. In some circumstances it may be sufficient for a care robot to provide the care recipient with a feeling of being respected and cared for. Second, this underscores that for care robots to be accepted, it may not be enough that they function in a technically correct and reliable manner, but they should also be able to express some kind of compassion and concern for those in need of care (see also 81).

### Limitations and Further Research Potential

We employed a quantitative research design to rigorously test our research hypotheses. This choice, however, comes at the cost of being unable to address the question of precisely why affective attitudes toward human and robot caregivers differ between actual care recipients and hypothetical future care recipients. Therefore, it is worthwhile to complement our quantitative study with qualitative research that could further investigate whether the observed differences are related to, for example, experience with caregiving situations or a greater perception of the usefulness of assistive technologies. This may reveal the exact triggers of differences in attitudes between actual care recipients and those who are not (yet) care-dependent.

A potential limitation of this study is also that we used a convenience participant sample. In 2019, the average age of care recipients in the US was 68.4 years, with a median age of 72 years [82]. Thus, although the median age of our sample was 44 years, and therefore higher than the median age of the US population, which was 38.4 years in 2019 [83], the participants were still largely younger than most care recipients are. Nevertheless, we chose to use a convenience sample due to the inaccessibility of elderly people in need of nursing care. Given the constraints of COVID-19 prevention restrictions in place at the time of our data collection, it was impossible to ensure comparable study conditions and perform a random treatment assignment in inaccessible nursing homes. However, as our sample included a substantial proportion of participants who reported needing nursing care at the time of the experiment, we are confident that our results are meaningful. Nonetheless, an interesting approach for future work would be to survey elderly people in nursing homes to assess their perceptions and experiences with assistive technologies and compare their responses to those of younger respondents who are not yet in need of care.

Furthermore, we conducted our study exclusively with US citizens and did not differentiate between different ethnic groups. However, culture has been found to influence not only people’s health, but also the quality of communication (e.g., in patient–physician encounters) and care [84]. Similarly, views on aging (or "anti-aging") and care of the elderly differ across countries, cultures, and ethnicities (e.g., in terms of whether supporting and caring for the elderly is seen as a societal, family, or individual responsibility) [85]. Previous research has further revealed the significant influence of individuals’ cultural background on their attitudes toward robots, their interactions with them, their acceptance of and preference for a particular appearance of robots, and the application domains and tasks conceivable to them (see, for instance, [84] for a recent literature review). These country-specific differences may stem from different belief systems and motivations, but also from different experiences with and exposure to robots [86]: While Western culture (and pop culture) often conveys a vision of doom in which robots—particularly evil robots—will take over the world, this is less common in Japanese culture, for example [87]. Cultural differences related to aging, elder care, and attitudes toward technology in general and robots in particular may also influence how people perceive care scenarios involving human and robot caregivers. Therefore, examining the interaction between these factors and affective attitudes would open opportunities for further promising research.

## Conclusion

Taken together, our results suggest the following. First, care-dependent people are less averse to care robots than is often assumed. Second, the attitudes of people toward robots in general and to the social aspects of human–robot interaction in particular play an essential role in their sense of comfort with care robots. Third, care robots’ acceptance will demand not only their technically correct function and reliability but also characteristics related to social inter*personal* interaction, such as appearing benevolent and respectful toward the person being cared for.

Through the systematic quantitative assessment of participants’ perceived comfort, we were able to concretely elicit the differences in affective attitudes of care-dependent and non-care-dependent participants when confronted with a care robot. Consequently, it is of the utmost importance that those who are not yet in need of care themselves be informed about the ways in and extent to which their own views and perceptions differ from the perceptions of those who would be affected most by using this technology. Our study, thus, supports the notion that ethical advice to policymakers should not be based solely on the introspective attitudes of ethicists but should systematically focus on the attitudes of the population (see, for instance, 88–90). Ethicists and policymakers, like any other individuals, may tend to project their current affective state onto their future state and, thus, underestimate how much their own views may change if they themselves become care-dependent at some point in the future [58]. Closing this empathy gap is difficult. This makes it crucial to elicit and understand the attitudes of those who will be directly affected by any given technology [64]. This task is particularly important if these technological beneficiaries’ attitudes reflect much less pronounced reservations about the use of new technologies. Future technologies’ ethical quality, moreover, should be measured by their potential to improve an existing situation rather than exclusively by a potentially unattainable ideal. For such a realistic assessment, the involvement of those affected proves particularly valuable. A better understanding of care recipients’ perception of potentially useful care technologies could promote those technologies’ use and thereby alleviate the societal problem of caregiver undersupply.

## Supplementary Information

Below is the link to the electronic supplementary material.

Supplementary file1 (DOCX 67 KB)

## Data Availability

Upon request from corresponding author.

## Footnotes

1 “Activities of daily living” refers to recurring activities that fulfill basic physical and psychological human needs and range from basic self-care tasks (such as personal hygiene maintenance, eating and drinking, and functional mobility [e.g., getting in and out of bed]) to instrumental activities required for independent living (such as shopping, taking medication, managing finances, and preparing meals) [4].

2 That is, if the use of robots does not represent an opportunity to remain living in one’s own home.

3 Issues with these two items (7: “The word ‘robot’ means nothing to me” and 14: “I feel that in the future, society will be dominated by robots”) are consistent with the results of other studies that have used NARS in Western societies (72–74). Pochwatko et al. [72], for instance, concluded that item 7 may be outdated, while item 14 may be culturally biased due to the different levels of robot exposure in different countries, which could lead to differing perceptions of the possible future role of robots in society.

4 The data of one participant who did not respond to any of the NARS items was removed from the data set for this part of the analysis.
