# Supplementary material for: The Care-Dependent are Less Averse to Care Robots: An Empirical Comparison of Attitudes
Source: Int J Soc Robot. 2023 May 29:1–18. Online ahead of print. doi: 10.1007/s12369-023-01003-2 (PMC10226445; doi:10.1007/s12369-023-01003-2)
Supplement: Supplementary file 1 — Supplementary file1 (DOCX 67 KB) [file 12369_2023_1003_MOESM1_ESM.docx]

Appendix A: Questionnaire

1. **Introduction**

*All participants read the following introduction:*

| Welcome to this questionnaire!  We are interested in your views on various situations in nursing care. For this purpose, we will first present you with two short descriptions of the situation and then ask you some questions. Please read the texts carefully and answer the questions conscientiously and truthfully.  Answering the questionnaire will take about 20 minutes.  Thank you in advance for your participation! |
| --- |

1. **Experimental Part**

*After reading the introduction, participants were randomly assigned to one of four conditions: H0, H25, R0 or R25. Each participant was introduced to the vignettes and subsequently read two vignettes: One dealt with an intimate care scenario, in which the caregiver helps with personal hygiene and one with a non-intimate service task, in which the caregiver brings the participants something to drink. The order of the vignettes was randomized. For both vignettes, participants were asked to rate their perceived level of comfort in the described situation (see 2.3).*

- 1. **Condition: Human Caregiver**
     1. **Introduction to vignettes**

**Condition H0**

| Please put yourself in the following situation:  Due to a physical illness, you will unexpectedly be in need of nursing care tomorrow and from then on you will need support in various activities of daily living. Therefore, you will move at short notice from your previous place of residence to a care facility. In this facility, human caregivers and care robots share the work.  A human caregiver is responsible for your ward. |
| --- |
| In the following we are particularly interested in your attitude toward the **human caregiver.** |

**Condition H25**

| Please put yourself in the following situation:  Due to a physical illness, you will unexpectedly be in need of nursing care in 25 years and from then on you will need support in various activities of daily living. Therefore, you will move at short notice from your previous place of residence to a care facility. In this facility, human caregivers and care robots share the work.  A human caregiver is responsible for your ward. |
| --- |
| In the following we are particularly interested in your attitude toward the **human caregiver**. |

- - 1. **Vignettes**

**Intimate scenario**

| In the following we are particularly interested in your attitude toward the human caregiver. |
| --- |
| One month after moving into the nursing home, you are in your room at 9 am, just like every day at this time, when there is a knock at your door. You invite in. **The human caregiver responsible for your ward** on this morning enters your room and asks you if he*she may **help you with your personal hygiene as usual**. You answer in the affirmative.  Since you have difficulty keeping your balance in the bathroom, you are washed in bed. The caregiver places your toothbrush on your usual place on the bedside table. He*She asks you about your preferred body care products and the desired water temperature. You brush your teeth. Meanwhile, the caregiver prepares the necessary care products and towels for washing, helps you to undress and hands you a damp washcloth. You wash your face and upper body yourself. The caregiver then continues with the personal hygiene. He*she turns you on your side and washes your back and buttocks. Then the caregiver washes your legs and intimate area.  After washing, the caregiver helps you to get dressed again and to sit comfortably in bed. He*She puts the used care products and towels back into their usual place in the bathroom.  The caregiver says goodbye and leaves your room. |

**Non-intimate scenario**

| In the following we are particularly interested in your attitude toward the human caregiver. |
| --- |
| One month after moving into the nursing home, you are in your room at 2 pm, just like every day at this time, when there is a knock at your door. You invite in. **The human caregiver responsible for your ward** on this afternoon enters your room and asks if he*she **may bring you something to drink, as usual at this time of day**. You answer in the affirmative.  Since you have difficulty keeping your balance when carrying objects, the caregiver brings a drinking glass and a water bottle on a tray to your bed. He*She places the tray on your usual place on the bedside table. He*She pours you some water and hands you the glass. You take a sip. The caregiver reminds you that you have drunk very little on this day so far and suggests that you might drink a little more. You realize that this is true and take another sip. Then, you put the glass down on your bedside table.  The caregiver helps you to sit comfortably in bed.  He*She says goodbye and leaves your room. |

- 1. **Condition: Care robot**
     1. **Introduction to vignettes**

**Condition R0**

| Please put yourself in the following situation:  Due to a physical illness, you will unexpectedly be in need of nursing care tomorrow and from then on you will need support in various activities of daily living. Therefore, you will move at short notice from your previous place of residence to a care facility. In this facility, human caregivers and care robots share the work.  A care robot is responsible for your ward. |
| --- |
| In the following we are particularly interested in your attitude toward the **care robot.** |

**Condition R25**

| Please put yourself in the following situation:  Due to a physical illness, you will unexpectedly be in need of nursing care in 25 years and from then on you will need support in various activities of daily living. Therefore, you will move at short notice from your previous place of residence to a care facility. In this facility, human caregivers and care robots share the work.  A care robot is responsible for your ward. |
| --- |
| In the following we are particularly interested in your attitude toward the **care robot**. |

- - 1. **Vignettes**

**Intimate scenario**

| In the following we are particularly interested in your attitude toward the care robot. |
| --- |
| One month after moving into the nursing home, you are in your room at 9 am, just like every day at this time, when there is a knock at your door. You invite in. **The care robot responsible for your ward** enters your room and asks if it may **help you with your personal hygiene as usual**. You answer in the affirmative.  Since you have difficulty keeping your balance in the bathroom, you are washed in bed. The care robot puts your toothbrush on its usual place on the bedside table. It asks you about your preferred body care products and the desired water temperature. You brush your teeth. Meanwhile, the caregiver prepares the necessary care products and towels for washing, helps you to undress and hands you a damp washcloth. You wash your face and upper body yourself. The care robot then continues with the personal hygiene. It turns you on your side and washes your back and buttocks. Then the care robot washes your legs and intimate area.  After washing, the care robot helps you to get dressed again and to sit comfortably in bed. It puts the used care products and towels back into their usual place in the bathroom.  The care robot says goodbye and leaves your room. |

**Non-intimate scenario**

| In the following we are particularly interested in your attitude toward the care robot. |
| --- |
| One month after moving into the nursing home, you are in your room at 2 pm, just like every day at this time, when there is a knock at your door. You invite in. The **care robot responsible for your ward** on this afternoon enters your room and asks if it may **bring you something to drink, as usual at this time of day**. You answer in the affirmative.  Since you have difficulty keeping your balance when carrying objects, the care robot brings a drinking glass and a water bottle on a tray to your bed. It places the tray on your usual place on the bedside table. It pours you some water and hands you the glass. You take a sip. The care robot reminds you that you have drunk very little on this day so far and suggests that you might drink a little more. You realize that this is true and take another sip. Then, you put the glass down on your bedside table.  The care robot helps you to sit comfortably in bed.  It says goodbye and leaves your room. |

**Rating of perceived comfort level**

*After reading the respective scenario, participants were asked to rate their perceived level of comfort in the described situation on a 7-point Likert scale.*

| Please indicate the degree to which you agree/disagree with the following statement. | Completely disagree | Largely disagree | Slightly disagree | Neither disagree nor agree | Slightly agree | Largely agree | Completely agree |
| --- | --- | --- | --- | --- | --- | --- | --- |
| I feel comfortable in the described situation. |  |  |  |  |  |  |  |

1. **Questionnaires**

*After completing the experimental part of the study, all participants were shown the following questionnaires*

- 1. Negative Attitudes toward Robots Scale (NARS)*

| Please indicate the degree to which you agree/disagree with the following statements. | | Strongly disagree | Disagree | Undecided | Agree | Strongly agree |
| --- | --- | --- | --- | --- | --- | --- |
| 01 | I would feel uneasy if robots really had emotions. |  |  |  |  |  |
| 02 | Something bad might happen if robots developed into living beings. |  |  |  |  |  |
| 03 | I would feel relaxed talking with robots. ****** |  |  |  |  |  |
| 04 | I would feel uneasy if I was given a job where I had to use robots. |  |  |  |  |  |
| 05 | If robots had emotions, I would be able to make friends with them. ****** |  |  |  |  |  |
| 06 | I feel comforted being with robots that have emotions. ****** |  |  |  |  |  |
| 07 | The word “robot” means nothing to me. *** |  |  |  |  |  |
| 08 | I would feel nervous operating a robot in front of other people. |  |  |  |  |  |
| 09 | I would hate the idea that robots or artificial intelligences were making judgments about things. |  |  |  |  |  |
| 10 | I would feel very nervous just standing in front of a robot. |  |  |  |  |  |
| 11 | I feel that if I depend on robots too much, something bad might happen. |  |  |  |  |  |
| 12 | I would feel paranoid talking with a robot. |  |  |  |  |  |
| 13 | I am concerned that robots would be a bad influence on children. |  |  |  |  |  |
| 14 | I feel that in the future, society will be dominated by robots. *** |  |  |  |  |  |

*Note. * Order of items was randomized. **Item was reverse coded for analysis. *** Item was removed from analysis.*

- 1. Need for Nursing Care

| *Intro:* **Now we would like to ask you some questions about the topic "need for nursing care".** | | |
| --- | --- | --- |
|  |  | |
| **Item** | | **Scale** |
| Are you in need of nursing care? | | - Yes - No |

- 1. Sociodemographic Questions

| *Intro:* **Finally, we would like to ask you for some personal details.** | |
| --- | --- |
|  | |
| **Item** | **Scale** |
| What is your age? | *[Please enter a number]* |
| What is your gender? | - Male - Female - Other |

Appendix B: Descriptive Statistics and Results of Non-Parametric Tests

Table B.1

Non-parametric test results of comfort ratings including the temporal distance of the vignettes.

|  | | | | *p*-value |
| --- | --- | --- | --- | --- |
| **Temporal Distance** | | | |  |
|  | Tomorrow vs. In 25 Years | | | 0.246 |
| **Caregiver** $\boldsymbol{\times}$ **Temporal Distance** | | | |  |
|  | Human | Tomorrow vs. In 25 Years | | 0.678 |
|  | Robot | Tomorrow vs. In 25 Years | | 0.159 |
| **Caregiver** $\boldsymbol{\times}$ **Scenario** $\boldsymbol{\times}$ **Temporal Distance** | | | |  |
|  | Human | Care | Tomorrow vs. In 25 Years | 0.751 |
|  | Human | Service | Tomorrow vs. In 25 Years | 0.899 |
|  | Robot | Care | Tomorrow vs. In 25 Years | 0.246 |
|  | Robot | Service | Tomorrow vs. In 25 Years | 0.529 |

Table B.2

Comfort and non-parametric test results depending on participants’ care dependency status.

|  |  | |  | | **Not Care-Dependent** | | | |  | **Care-Dependent** | | | | |  | **Non-parametric test results** | |
| --- | --- | --- | --- | --- | --- | --- | --- | --- | --- | --- | --- | --- | --- | --- | --- | --- | --- |
|  |  | |  | | N | Mean | | SD |  | N | | | Mean | SD |  | *p*-value | d |
| **Care Dependency** | | | | | | | | | | | | | | | | | |
|  |  | |  | | 1,159 | 3.97 | | 1.54 |  | 114 | | | 4.89 | 1.29 |  | <0.001 | -0.612 |
| **Caregiver** $\boldsymbol{\times}$ **Care Dependency** | | | | | | | | | | | | | | | | | |
|  | Human | |  | | 578 | 4.34 | | 1.24 |  | 57 | | | 4.84 | 1.25 |  | <0.001 | -0.401 |
|  | Robot | |  | | 581 | 3.59 | | 1.70 |  | 57 | | | 4.95 | 1.35 |  | <0.001 | -0.810 |
| **Caregiver** $\boldsymbol{\times}$ **Scenario** $\boldsymbol{\times}$ **Care Dependency** | | | | | | | | | | | | | | | | | |
|  | Non-Intimate | Human | |  | 578 | 4.84 | | 1.28 |  | 57 | | | 4.86 | 0.76 |  | 0.900 | -0.013 |
|  |  | Robot | |  | 581 | 3.97 | | 1.09 |  | 57 | | | 5.00 | 0.66 |  | <0.001 | -0.583 |
|  | Intimate | Human | |  | 578 | 3.85 | | 1.28 |  | 57 | | | 4.82 | 0.76 |  | <0.001 | -0.572 |
|  |  | Robot | |  | 581 | 3.22 | | 1.09 |  | 57 | | | 4.89 | 0.66 |  | <0.001 | -0.886 |
|  |  | |  | |  |  | |  |  |  | | |  |  |  |  |  |
| **Caregiver** $\boldsymbol{\times}$ **Care Dependency** | | | | | | | | | | | | | | | | | |
|  | Not Care-Dependent | | Human vs. Robot | | | | |  |  |  | | |  |  |  | <0.001 | 0.505 |
|  | Care-Dependent | | Human vs. Robot | | | | |  |  |  | | |  |  |  | 0.338 | -0.081 |
| **Caregiver** $\boldsymbol{\times}$ **Scenario** $\boldsymbol{\times}$ **Care Dependency** | | | | | | | | | | | | | | | | | |
|  | Not Care-Dependent | | Non-Intimate | | | | Human vs. Robot | | | |  |  |  |  |  | <0.001 | 0.559 |
|  |  | | Intimate | | | | Human vs. Robot | | | |  |  |  |  |  | <0.001 | 0.341 |
|  | Care-Dependent | | Non-Intimate | | | | Human vs. Robot | | | |  |  |  |  |  | 0.261 | -0.101 |
|  |  | | Intimate | | | | Human vs. Robot | | | |  |  |  |  |  | 0.766 | -0.050 |
